# A personalised screening strategy for diabetic retinopathy: a cost-effectiveness perspective

**DOI:** 10.1007/s00125-020-05239-9

**Published:** 2020-07-31

**Authors:** Sajad Emamipour, Amber A. W. A. van der Heijden, Giel Nijpels, Petra Elders, Joline W. J. Beulens, Maarten J. Postma, Job F. M. van Boven, Talitha L. Feenstra

**Affiliations:** 1 Department of Clinical Pharmacy and Pharmacology, University Medical Center Groningen, University of Groningen, 9700 RB Groningen, the Netherlands; 2 Department of General Practice and Elderly Care Medicine, Amsterdam University Medical Center, location VU, Amsterdam, the Netherlands; 3 Department of Health Sciences, University Medical Center Groningen, University of Groningen, Groningen, the Netherlands; 4 Faculty of Science and Engineering, Groningen Research Institute of Pharmacy, University of Groningen, Groningen, the Netherlands; 5 Department of Economics, Econometrics & Finance, Faculty of Economics & Business, University of Groningen, Groningen, the Netherlands; 6 Department of Epidemiology, University Medical Center Groningen, University of Groningen, Groningen, the Netherlands; 7 National Institute for Public Health and the Environment (RIVM), Bilthoven, the Netherlands

**Keywords:** Cost-effectiveness, Diabetic retinopathy, Risk assessment, Screening intervals

## Abstract

**Aims/hypothesis:**

In this study we examined the cost-effectiveness of three different screening strategies for diabetic retinopathy: using a personalised adaptive model, annual screening (fixed intervals), and the current Dutch guideline (stratified based on previous retinopathy grade).

**Methods:**

For each individual, optimal diabetic retinopathy screening intervals were determined, using a validated risk prediction model. Observational data (1998–2017) from the Hoorn Diabetes Care System cohort of people with type 2 diabetes were used (*n* = 5514). The missing values of retinopathy grades were imputed using two scenarios of slow and fast sight-threatening retinopathy (STR) progression. By comparing the model-based screening intervals to observed time to develop STR, the number of delayed STR diagnoses was determined. Costs were calculated using the healthcare perspective and the societal perspective. Finally, outcomes and costs were compared for the different screening strategies.

**Results:**

For the fast STR progression scenario, personalised screening resulted in 11.6% more delayed STR diagnoses and €11.4 less costs per patient compared to annual screening from a healthcare perspective. The personalised screening model performed better in terms of timely diagnosis of STR (8.8% less delayed STR diagnosis) but it was slightly more expensive (€1.8 per patient from a healthcare perspective) than the Dutch guideline strategy.

**Conclusions/interpretation:**

The personalised diabetic retinopathy screening model is more cost-effective than the Dutch guideline screening strategy. Although the personalised screening strategy was less effective, in terms of timely diagnosis of STR patients, than annual screening, the number of delayed STR diagnoses is low and the cost saving is considerable. With around one million people with type 2 diabetes in the Netherlands, implementing this personalised model could save €11.4 million per year compared with annual screening, at the cost of 658 delayed STR diagnoses with a maximum delayed time to diagnosis of 48 months.

**Graphical abstract:**

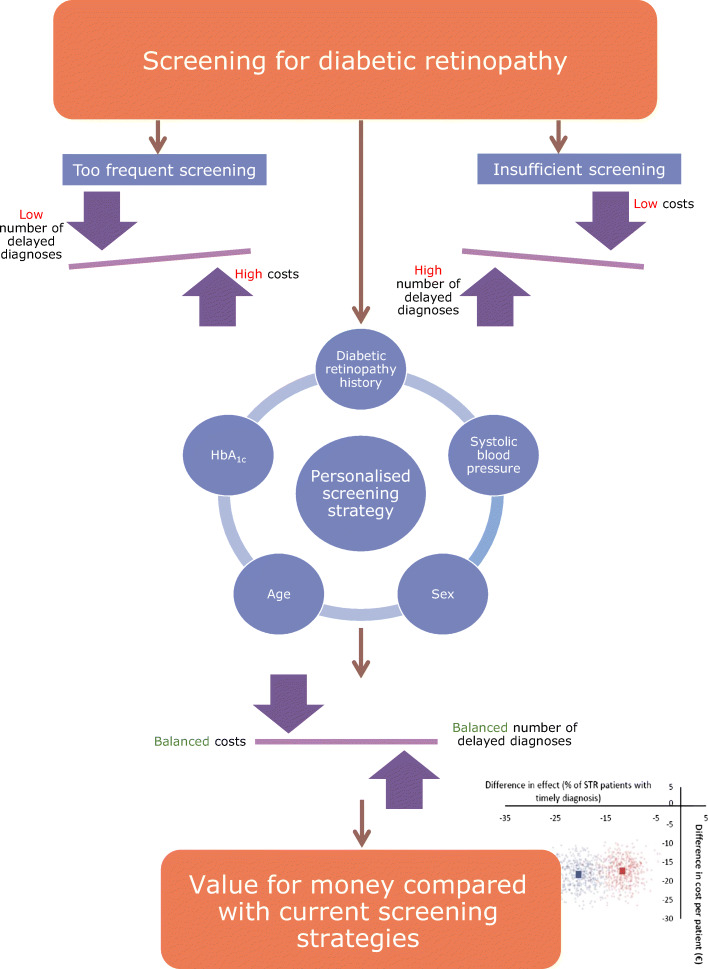

**Electronic supplementary material:**

The online version of this article (10.1007/s00125-020-05239-9) contains peer-reviewed but unedited supplementary material, which is available to authorised users.

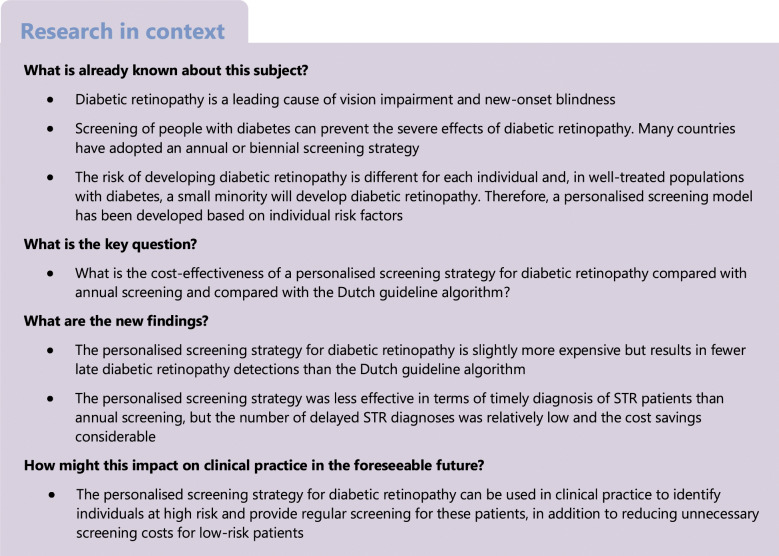



## Introduction

Diabetic retinopathy is the leading cause of vision impairment and new-onset blindness in many countries [1–3]. It has been estimated that the global prevalence of diabetes among adults will increase from 8.4% in 2017 to 9.9% in 2045 (from 451 million to 693 million people, age 18–99 years) [4]. Hence, more people may be affected by diabetic retinopathy. In the Netherlands, the population with diabetes was estimated at 1.1 million, of which 90% had type 2 diabetes [5], and around 10–15% of all people with diabetes had diabetic retinopathy [6]. Since detecting diabetic retinopathy in early stages can prevent severe irreversible damage [7], screening for diabetic retinopathy in people with diabetes is vital. Therefore, in many countries, either annual or biennial (every 2 years) screening is recommended [8, 9]. Such screening serves to refer individuals with diabetic retinopathy to secondary care in time to prevent or limit such severe damage. Internationally, guidelines vary in the level of retinopathy indicated for a referral to an ophthalmologist for immediate treatment or careful follow-up. In the current Dutch guidelines, as in the UK, the EURODIAB grade 3 (UK R2 level; sometimes also referred to as sight-threatening retinopathy [STR]) is considered an appropriate level for referral [6].

However, not all people with diabetic retinopathy develop STR. In those that do develop STR, a latent stage of diabetic retinopathy, symptoms are clearer and have a significant impact on patients’ quality of life [10, 11], and usually require active treatment. Notably, the risk of developing STR is highly variable and ‘one size fits all’ approaches may therefore not only lead to late screening in high-risk patients, but also over-screening in low-risk patients [12, 13]. Moreover, from a health economics perspective, ‘one size fits all’ approaches will pose unnecessary costs for both healthcare systems and patients.

In previous cost-effectiveness analyses of diabetic retinopathy screening, different screening algorithms have been explored. Some algorithms assumed variable screening intervals by stratifying patients varying from 6 months up to 5 years [14, 15]. A recent review of diabetic retinopathy prediction models showed the models by Scanlon et al [15] and Aspelund et al [12] performed better than other models [16]. Scanlon et al developed a personalised risk profile in which the screening intervals varied from 6 months to 5 years; the cost-effectiveness of this model has been previously assessed [15]. Aspelund et al proposed a model to compute personalised screening intervals based on individual patient characteristics [12]. This model was validated in different cohorts, including the cohort used in the present study, showing good model performance [13, 16–18]. Nevertheless, the cost-effectiveness of this screening strategy has not been assessed. In the Dutch guideline, renewed in 2016, a stratified approach with screening intervals ranging from 1 to 3 years was advised, based on previous presence of retinopathy. In the present study we aimed to assess the cost-effectiveness of a personalised screening interval based on estimated diabetic retinopathy risk using the Aspelund model compared with screening strategies using a fixed interval (annual screening) and a stratified approach based on previous retinopathy grades (Dutch guideline).

## Methods

### Study design

We performed a cost-effectiveness study based on routine care data. After optimising the personalised strategy by setting its risk margin, results for personalised and Dutch guideline screening strategies were compared with annual screening in terms of differences in costs and the number of delayed STR diagnoses. Then, a comparison between the personalised and Dutch guideline screening strategies was performed.

### Study population

To study the impact of different diabetic retinopathy screening intervals on development of STR, we used the Hoorn Diabetes Care System (DCS) cohort, a dynamic primary care cohort of people with type 2 diabetes in the Netherlands [19]. Data were available over the period from the beginning of 1998 up to the end of 2017. There were 13,959 people in the total cohort, with a varying date of entry and exit, resulting in patient follow-up varying between 0 and 18 years. Inclusion criteria for the current study were: (1) at least one measurement of a grade of retinopathy available; (2) having no STR at baseline; and (3) at least 5 years of follow-up available. The latter criterion was based on the maximal screening interval that the Aspelund et al model would predict, i.e. 5 years [12]. For all participants, we used patient-level results from all routine clinical and laboratory measurements that were available. The measurements in the cohort included, but were not limited to, systolic BP (SBP), diastolic BP, HbA_1c_, BMI, cholesterol level and triacylglycerol, date of diagnosis of type 2 diabetes, ethnic group, and fundus photography including retinopathy grades. The retinopathy grades were reported according to the EURODIAB scale, which ranges from 0 to 5 [20]: grade 0 means no retinopathy; grade 1 is ‘minimal non-proliferative retinopathy’; grade 2 is ‘moderate non-proliferative retinopathy’; grade 3 is ‘severe non-proliferative retinopathy’; grade 4 is ‘photocoagulated retinopathy’; and grade 5 is ‘proliferative retinopathy’. According to the Dutch guideline, grades 3–5 were considered STR, and usually, patients with these grades were referred to an ophthalmologist for treatment [13, 16]. The study has been approved by the Medical Ethical Review Committee of the VU University Medical Center, Amsterdam. Individuals were informed about the use of their data and were offered an opt-out. Data were used anonymously.

### Imputation for missing values

Missing explanatory variables (duration of diabetes, HbA_1c_ and SBP) were imputed by the mean value conditional on retinopathy grade. For missing values of the dependent variable of retinopathy grade, a different approach was followed. First, we used information from adjacent measurements in the same individual for interpolation, and if the grades before and after the missing value were the same, we filled out the missing value with that grade. If grades differed, a uniform distribution between the two adjacent grades was assumed, and a random draw from this distribution was applied and rounded to an integer value. Second, for patients with STR missing the time to develop STR, two extreme scenarios were used to reflect a broad range of possible outcomes, i.e. slow and fast STR progression assumptions. In the slow STR progression assumption, STR was assumed to have developed at the last possible time point. In the fast STR progression assumption, STR was assumed to have developed at the first possible time point. Fast STR progression was taken as a base assumption for imputation and slow STR progression assumption used for sensitivity analysis.

Duration of diabetes, HbA_1c_ and SBP were missing for 1.0%, 1.7% and 2.0%, respectively, of all records. Retinopathy grades of participants without STR showed 12.2% missing values over all the records. Of these, 10.1% had the same grades before and after the missing value, while for the remaining 2.1%, grades differed. For 22% of participants with STR, some retinopathy grades were missing, and needed to be imputed.

### Data analysis

In order to perform cost-effectiveness analysis, first, we needed to optimise the screening model [12] to determine the personalised diabetic retinopathy screening strategy. To do so, a preset risk margin (i.e. the risk of developing STR given a certain screening interval) was assumed. Subsequently, different intervals using a personalised diabetic retinopathy screening model were simulated, and the clinical outcomes and costs of these screening intervals were calculated.

### Determining personalised screening intervals

To estimate personalised screening intervals, we used the model previously developed by Aspelund et al [12]. A brief description of Aspelund’s model is provided in electronic supplementary material (ESM) Methods. In short, this model predicts the time to develop STR based on individual patient characteristics which include mean blood glucose or HbA_1c_, SBP, presence of retinopathy, sex, and duration of diabetes. In the original publication, the personalised intervals for screening using Aspelund’s model were determined based on a preset fixed risk margin of 3.2%. This risk margin was derived from the proportion of people that developed STR in their first year of the screening programme in the original dataset used by Aspelund et al [12], and basically reflected patients’ current real-world STR risk. Of note, in our analysis, we varied this risk margin between 0.0% and 4.0% and applied the corresponding personalised screening interval in our simulation. Before comparison with the other two strategies, we optimised the risk margin from the perspective of costs per case of delayed diagnosis, as explained below.

### One to 3 year screening interval recommended by the Dutch guideline

In the Dutch diabetic retinopathy guideline, people with diabetes are divided into two subgroups: those without previously known retinopathy and those already having a low retinopathy grade at baseline. The recommended diabetic retinopathy screening intervals are based on the retinopathy grade. If the individual does not have retinopathy at the first visit, then the next screening visit is after 2 years. If after 2 years still no retinopathy is present, the screening interval is increased to 3 years. If the individual has mild retinopathy (grades 1–2), then the screening interval will be annual, and in severe cases (grades 3–5) patients should be referred to an ophthalmologist [6].

### Clinical outcomes

The clinical outcome was the number of delayed STR diagnoses. This was calculated by counting the number of patients for whom the personalised screening model that was simulated predicted longer intervals than the observed time to STR diagnosis (based on the real-world longitudinal DCS cohort follow-up). In the present study, we did not consider quality-adjusted life years (QALYs) as an outcome since an estimate of QALYs would require elaborate modelling which would introduce more uncertainty in the study.

### Costs

We considered the healthcare perspective for the main cost analysis, while we added productivity losses and travel costs in a sensitivity analysis. Discount rates of 1.5% and 4.0% were applied to effects and costs, respectively, in line with Dutch health economic guideline recommendations [21]. We did not include treatment costs, since all patients with retinopathy will eventually be treated in each of the three different strategies, with only slight differences in the timing of treatment. Price levels used were for 2015.

For the maximum screening costs, the tariff of a large Dutch commercial laboratory was used [22]. For the minimum screening costs, a calculation based on micro-costing was applied [23]. Screening costs ranged from €15.25 to €41.07. Travel costs ranged from €1.58 to €14.19 [23]. Productivity losses were estimated to vary from €2.63 to €16.62 [24]. For all cost types, a gamma distribution was fitted to the minimum and maximum cost estimates to reflect the uncertainty. The details of costs are shown in ESM Table 1.

### Determination of the best risk margin for personalised screening

To determine the best risk margin for use with the Aspelund model, risk margins were varied from 0 to 4, and for each risk margin the savings per case with a delayed STR diagnosis were assessed using a stepwise approach. For each 0.1% increase in the risk margin, we calculated the incremental cost saving and compared these to the additional delayed STR diagnoses. The incremental cost saving per delayed STR diagnosis was computed as the ratio of differences in costs to differences in delayed STR diagnoses for each risk margin as compared with the previous risk margin. The best risk margin was considered to be the one with the lowest number of delayed STR cases at which incremental savings per delayed STR case would peak.

### Sensitivity analysis

In order to capture the uncertainty around the study sample and around the costs of screening, we conducted bootstrapping (1000 simulations) from the study sample and varied costs over their full ranges.

Bootstrapping with 1000 simulations was performed for the fast and slow progression assumptions. In the first step, the best risk margin in each iteration was determined. Then, for the mean over the iterations of the best risk margin, probabilistic sensitivity analysis using different cost estimates for healthcare perspective (screening cost) and societal perspective (travel cost and productivity loss) was conducted with 1000 iterations, again varying the sample using bootstrapping techniques, but now also varying the costs.

All analyses were performed with the statistical software package R (version 3.6.1) (www.rproject.org) [25] in combination with Microsoft Excel 2010 for Windows.

## Results

### Study population selection and characteristics

**Fig. 1:**
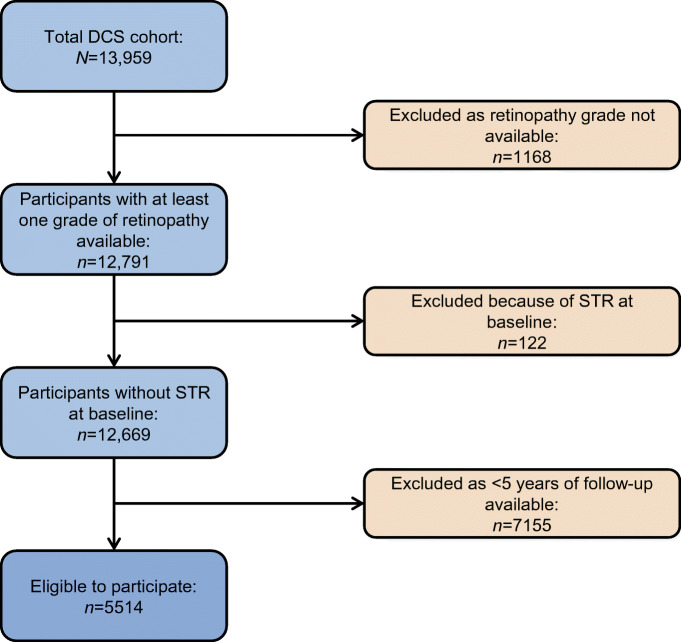
Patient selection flow chart

In the DCS cohort, at least one grade of retinopathy was available for 12,791 people with diabetes. Among these, 122 patients had STR (grade 3, 4 or 5) at baseline and were excluded. Figure 1 shows the flow chart of participant selection and how many were excluded in each step.

Study population characteristics are shown in Table 1 by retinopathy grade at baseline. On average, as the retinopathy grade increased, the levels of SBP, HbA_1c_ and duration of diabetes at baseline also increased.

**Table 1:** Baseline characteristics of the study population

Characteristic	No retinopathy	Grade 1 retinopathy	Grade 2 retinopathy
Retinopathy grade, *n*, %	5173 (93.8)	282 (5.1)	59 (1.1)
Age, mean (SD)	60.3 (10.8)	61.2 (10.8)	60.0 (9.9)
Male, %	53.9	54.6	62.7
Diabetes duration, years, median (IQR)	0.8 (0.2–2.9)	2.11 (0.3–7.1)	5.8 (1.5–13.6)
HbA_1c_, mmol/mol, mean (SD)	55.7 (17.1)	60.9 (19.7)	70.4 (19.4)
HbA_1c_, %, mean (SD)	7.2 (1.5)	7.7 (1.8)	8.6 (1.8)
SBP, mean (SD)	142.5 (20.1)	145.2 (22.3)	149.1 (20.5)

### Number of delayed STR diagnoses for different risk margins

**Fig. 2:**
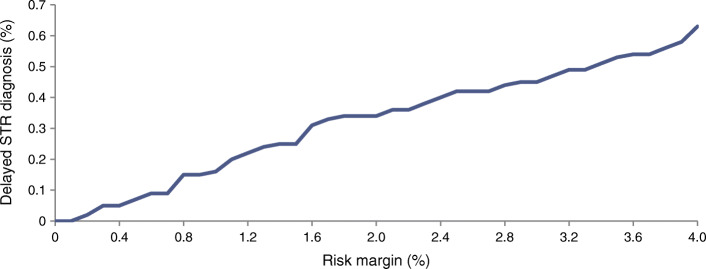
The percentage of delayed STR diagnoses for different risk margins for fast STR progression, mean over 1000 bootstrap replications

Figure 2 shows the percentage of delayed STR diagnoses based on the fast STR progression assumptions at different risk margins. Using the fast STR progression assumption, the number of delayed STR diagnoses varied from 0 (0.0%) to 35 (0.63%). The number of delayed STR diagnoses in the slow STR progression assumption is shown in ESM Fig. 1.

### Determining the best risk margin

**Fig. 3:**
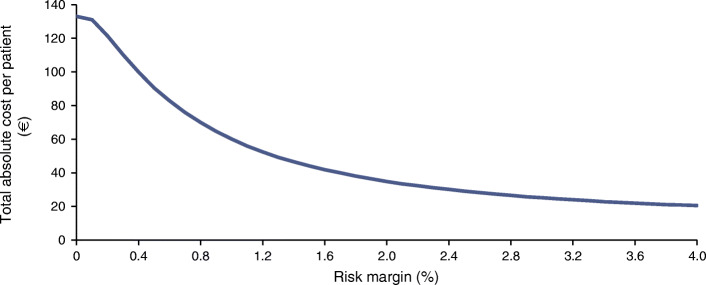
Total absolute costs of screening for different risk margins from a healthcare perspective with the fast STR progression assumption, mean over 1000 bootstrap replications

Figure 3 shows the total absolute cost of personalised screening (per patient) from a healthcare perspective. The cost started at €27 for a risk margin of 0.0% and decreased to €4 per patient per year for a risk margin of 4.0%. Very low risk margins implied more frequent screening, and hence as the risk margin increased, costs started to decline, but the number of delayed STR diagnoses rose (Fig. 2). Screening costs seemed to reach a plateau, which implies that, once high, increasing risk margins do not continue to bring many extra savings.

**Fig. 4:**
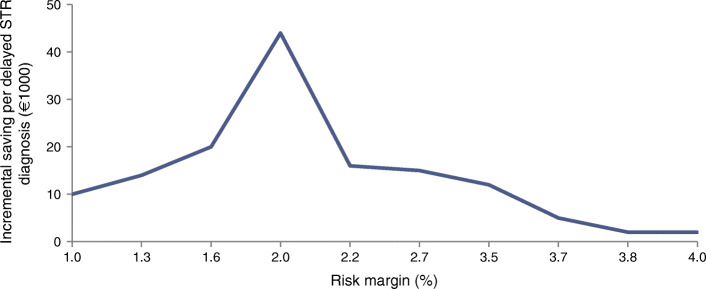
Incremental saving per delayed STR diagnosis for the fast STR progression assumption from a healthcare perspective, mean over 1000 bootstrap replications

Figure 4 combines Figs 2 and 3 and shows the incremental cost saving per delayed STR diagnosis, compared with a slightly lower risk margin from the healthcare perspective. It is important to note that increasing the risk margin decreased costs but increased the number of delayed STR diagnoses. The risk margin that maximised the incremental saving per delayed STR diagnosis for the slow progression assumption was the same as for the fast progression assumption and was 2.0% (ESM Fig. 2). The mean screening intervals started at 6.0 months and increased to 31.7 months for risk margins between 0.0% and 4.0%. The mean screening intervals and the number of delayed STR diagnoses for different risk margins and the best risk margin are shown in ESM Tables 2 and 3 for the slow and fast progression assumption.

### Costs and outcomes of personalised screening compared with the annual screening and Dutch guideline

**Fig. 5:**
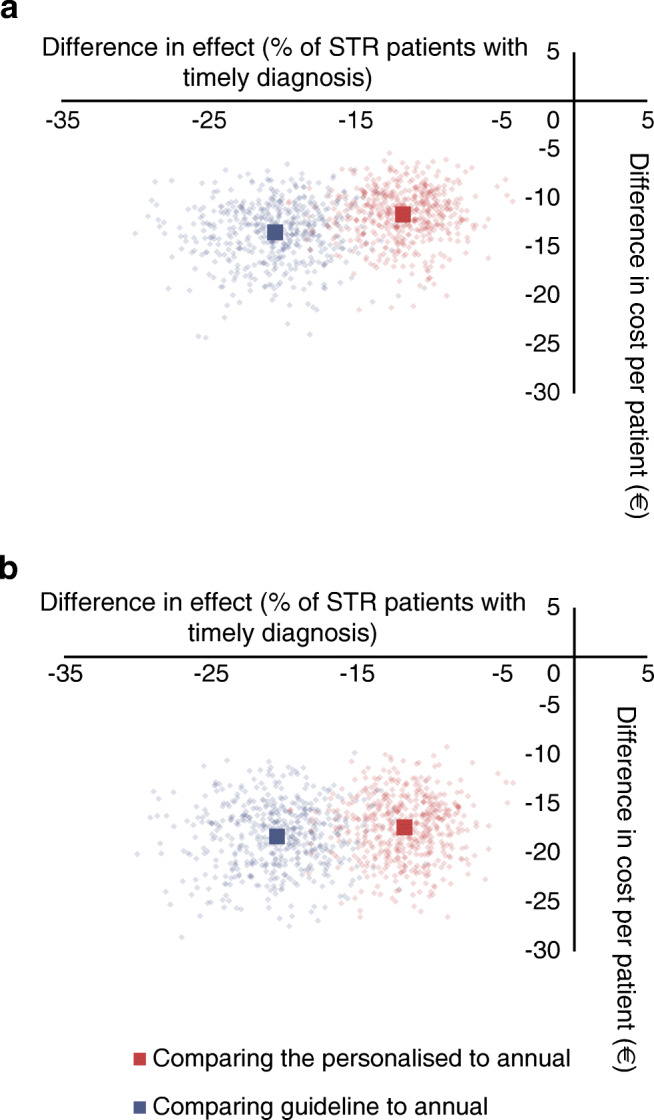
Cost-effectiveness plane with 1000 bootstrapping simulations for fast STR progression; (**a**) healthcare perspective and (**b**) societal perspective

The DCS cohort was mostly subject to annual screening as it covered the period 1998–2017. The personalised screening strategy resulted in 15 to 18 (9.7% to 11.6% of STR cases) delayed STR diagnoses, for the slow and fast STR progression assumptions, respectively. Applying a screening strategy according to the current Dutch guideline to this cohort would result in a total of 28 to 32 (18.1% to 20.7% of STR cases) delayed STR diagnoses, which is more than the personalised screening strategy, for the slow and fast STR progression assumptions, respectively. Figure 5 shows the cost-effectiveness plane of the personalised screening strategy compared with annual screening and the Dutch guideline screening strategy compared with annual screening for fast STR progression from a healthcare and societal perspective. The corresponding figure for slow STR progression is shown in ESM Figs 3 and 4. Both the personalised and the Dutch guideline screening strategy were less effective regarding delayed STR diagnoses but also less costly than the annual screening strategy. The Dutch guideline screening strategy was less effective and less costly than personalised screening for both fast and slow STR progression. Assuming fast progression, the median delay for the Dutch guideline strategy was 12.0 months while for the personalised model, the median delay was 15.3 months. Assuming slow STR progression, the median delay for the Dutch guideline strategy was 12.0 months, while for the personalised model this was 13.5 months. ESM Tables 3 and 4 show the mean, median and IQR of these delays and also the baseline grade for delayed STR diagnoses.

### Sensitivity analysis

The mean of best risk margins were 2.0% (95% CI 1.3%, 2.9%) and 2.0% (95% CI 1.3%, 3.1%) for the fast and slow progression assumptions, respectively. Table 2 shows the results of the sensitivity analyses in terms of total costs, saving per patient per year, incremental cost-effectiveness ratio, and the number of delayed STR diagnoses by comparing the personalised and Dutch guideline screening strategies with the annual screening strategy for a risk margin of 2.0% with the fast STR progression assumption. The results for slow STR progression assumption are presented in ESM Table 5. Both the personalised and the Dutch guideline screening strategy performed worse in terms of detecting STR cases on time, but they cost less compared with annual screening. While on average the personalised screening was slightly more expensive than the Dutch guideline, it could detect the STR cases more effectively.

**Table 2:** Total costs, saving, incremental cost-effectiveness ratio and number of delayed STR diagnoses for different strategies with a fast progression assumption

Item	Healthcare perspective (95% CI)	Societal perspective (95% CI)
Total costs – annual screening, €1000	682 (433, 966)	1018 (721, 1336)
Total costs – personalised screening, €1000	361 (228, 510)	539 (380, 717)
Total costs – Dutch guideline screening, €1000	310 (196, 437)	472 (325, 610)
Saving per patient per year – personalised compared with annual, €	11.4 (7.5, 16.5)	17.2 (12.4, 22.9)
Saving per patient per year – Dutch guideline compared with annual, €	13.2 (8.8, 19.1)	19.9 (14.3, 26.7)
ICER saving per delayed STR diagnosis – personalised compared with annual, €1000	18,844 (10,516, 32,533)	28,137 (16,500, 43,049)
ICER saving per delayed STR diagnosis – Dutch guideline compared with annual, €1000	12 (7, 19)	18 (11, 26)
Number of delayed STR diagnoses – personalised screening (out of 155 STR cases)	18.0 (11.5, 24.9)	18.0 (11.5, 24.9)
Number of delayed STR diagnoses – Dutch guideline screening (out of 155 STR cases)	31.6 (23.0, 41.3)	31.6 (23.0, 41.3)

ICER, incremental cost-effectiveness ratio

In order to compare different strategies, first the comparison of personalised screening and Dutch guideline with the annual screening was performed. The cost-effectiveness planes and cost-effectiveness acceptability curves (CEACs) are shown in ESM Figs 3 and 4 and ESM Fig. 5, respectively. In all the CEAC graphs, the probability of accepting personalised screening is higher than the Dutch guideline. Then the personalised screening was compared with Dutch guideline (ESM Figs 6, 7). In the CEAC curve the Dutch guideline had a relatively low probability of being cost-effective for most threshold values of saving per delayed STR diagnosis compared with personalised screening (ESM Fig. 8.)

## Discussion

### Main findings

Personalised screening for retinopathy using a risk prediction model developed by Aspelund et al and an optimised risk margin implied an increase of 11% in the number of delayed STR diagnoses, or a mean delay of 18 months compared with annual screening, resulting in cost savings of nearly €11 per patient per year from a healthcare perspective. Compared with the current Dutch guideline, for fast STR progression, the personalised screening strategy resulted in around 9% less delayed STR diagnoses, at a slightly higher cost per patient (€1.8), respectively, from a healthcare perspective. Of note, the mean delay in the personalised model was about 6 months longer than in the Dutch guideline algorithm. This delay was due to the larger maximum screening interval of 5 years, vs 3 years in the Dutch guideline. When personalised screening intervals were applied, the mean best risk margin for both assumptions was 2.0%. This risk margin is stricter than the risk margins that were proposed in the original publications of the risk prediction model.

### Interpretation

Screening for diabetic retinopathy is one of the most effective ways of preventing severe and irreversible complications. Annual screening has been recommended by WHO and most health institutes [12]. In line with our findings, a publication by Scanlon et al showed that annual screening is not cost-effective [15]. However, according to a systematic review by Taylor-Phillips et al, not enough evidence existed to show that extending the screening interval to more than 1 year is safe [26]. Instead of delayed detection of STR cases, Scanlon et al used QALYs as an outcome, and they concluded that for patients without retinopathy at baseline, 3 year interval-based screening was the most cost-effective strategy [15], while some other studies suggested a 2 to 3 year screening interval was cost-effective [27–29].

While more favourable from a cost-effectiveness perspective, a possible limitation of implementing a personalised model is a reduction in screening attendance, when variable intervals cause a changed engagement or changed routine. Within the DCS setting of annual diabetes control visits, with or without the retinopathy screening embedded, this risk is probably quite small. However, having variable screening intervals might have an impact on attendance rate [30].

Our current analysis adds a cost-effectiveness study of another personalised screening strategy, using the Aspelung prediction model, and comparing it to both annual screening and stratified (Dutch guideline) screening. From a clinical, epidemiological perspective, the original personalised model of Aspelund et al that was used in our analysis proposed a risk margin of 3.2%. Based on the ratio of extra savings to the number of delayed STR diagnoses, our evaluation indicated that stricter risk margins of 2.0% (95% CI 1.3%, 3.1%) performed better. This stricter risk margin also reduces the mean delay in detecting STR cases.

### Strengths and limitations

The strength of this study is that we used observational data from routine clinical practice for a large number of people with type 2 diabetes with longitudinal follow-up. However, thanks to routine care for people with diabetes in the Netherlands, the number of STR cases is relatively rare in the cohort. A limitation inherent to the use of routine care data is the presence of missing values. However, the proportion of missing values in the predictors was relatively low. Missing annual retinopathy grades, mostly as a result of clinicians already applying biennial screening intervals, were more problematic since these were the outcomes rather than the predictors and they were missing for 22% of the STR cases, and 2% of the non-STR cases. We addressed this by investigating a slow and fast STR progression assumption for STR and by interpolating missing values for non-STR cases. Findings for the fast STR progression assumption are to be considered as conservative since missing values were assumed to have the highest observed retinopathy grade.

The cohort used in this study is a well-controlled diabetes population with centrally organised care, and checks related to diabetes risk factors are performed on an annual basis. Therefore, generalising this model to less well-controlled diabetes populations should be done with caution. The grade of retinopathy in each eye was not available in the cohort; however, it will not have an impact on our analyses because in the Aspelund et al model, the overall grade is needed. While a delayed diagnosis will not improve the condition, due to the different risk factors involved in diabetic retinopathy, it remains unclear as to how much a delay puts the patients in danger of irreversible damage or even blindness. On the basis of experts’ opinions, the consequences of an STR diagnosis delayed up to 2 years may not have an impact on patients’ conditions. However, we do not know the exact clinical and economic consequences of late diagnosis, and therefore, although relevant, these costs were not taken into account. Similarly, only effects in terms of delayed STR were compared rather than modelling the effects of delay over a lifetime horizon which would be needed to estimate QALYs as an outcome. Such long-term modelling would add more complexity and uncertainty in the study. Hence, the current study used delayed STR diagnosis as the health outcome of interest, instead of QALYs, which is in contrast to a previous study by Scanlon et al [15]. Costs per case detected late are sufficient to enable comparison of various screening strategies for their cost-effectiveness. However, when policymakers want to compare retinopathy screening to other diabetes treatments or broader healthcare policy, a cost per QALY outcome is required. Another limitation of this study was that we assumed that annual screening did not result in any delayed detection of STR cases since the minimum screening interval in the cohort between 1998 and 2013 was 1 year. The personalised strategy also permits 6 month screening intervals for the highest risk group and, as such, offers a higher level of surveillance for these individuals.

### Recommendations

Although the model proposed by Aspelund et al performed well on predicting the personalised diabetic retinopathy screening intervals based on patient risk factors and reduced total screening costs, still the proportion of delayed STR diagnoses was relatively high at 11%, indicating room for further improvement of the model. The maximum screening interval in this model is 5 years, leading to a median delay of 15.3 months. More knowledge is needed on the consequences of such delays, and what maximum delay in detecting STR is acceptable from a clinical point of view. This knowledge could be used to modify the maximum screening interval.

In conclusion, a personalised diabetic retinopathy screening strategy, in a well-controlled diabetes population, led to cost savings at a loss of health in terms of additional delay in STR detection for 11% of individuals with STR. At the same time, it outperformed the stratified approach based on current retinopathy grade as advocated in the Dutch guidelines. With around 1 million individuals with diabetes in the Netherlands, implementing this personalised model could save €11.4 million per year compared with annual screening, at the cost of 551 to 658 delayed diagnoses of STR.

## Electronic supplementary material

ESM 1 (PDF 548 kb)

## Data Availability

The steering committee of the Hoorn studies will consider reasonable requests for the sharing of de-identified patient-level data. Requests should be made to the corresponding author.

## Abbreviations

CEAC: Cost-effective acceptability curve
DCS: Hoorn Diabetes Care System
QALY: Quality-adjusted life years
SBP: Systolic BP
STR: Sight-threatening retinopathy
