# Supplementary material for: A personalised screening strategy for diabetic retinopathy: a cost-effectiveness perspective
Source: Diabetologia. 2020 Jul 31;63(11):2452–61. doi: 10.1007/s00125-020-05239-9 (PMC7527375; doi:10.1007/s00125-020-05239-9)
Supplement: Supplementary file 1 — (PDF 548 kb) [file 125_2020_5239_MOESM1_ESM.pdf]

Electronic supplementary material (ESM)

ESM Methods: Model for prediction of personalized screening intervals

In the model [1], first the baseline survival probability of no STR was calculated and then the personalized interval was estimated based on the characteristics of each patient. In the survival model, baseline STR risk was $S_0(t) = \exp(-\exp[-4.88]\, t^{1.17}) - 0.052$, where t is the duration of diabetes. For each individual patient, the survival function is $S(t) = S_0(t)^{\exp\,(linear\ combination)}$, and the linear combination is:

$$(HbA_{1c}[\%] - 8) \times 0.380544 + (SBP - 130) \times 0.04308 + (DRpresent - 0.33) \times (0.89 + \beta_{DR})$$

Where HbA1c is blood glucose, SBP is systolic blood pressure, DRpresent is 1 if the patient has retinopathy (grades 1 and 2) at baseline and otherwise 0, $\beta_{DR} = 0.46$ for men and $\beta_{DR} = -0.46$ for women if diabetic retinopathy is present and otherwise $\beta_{DR} = 0$. The risk of developing STR within a time interval Δt is then computed as:

$$risk(\Delta t | disease\ free\ at\ t) = 1 - S(t + \Delta t)/S(t)$$

Solving the above equation for Δt, for a given risk margin r between 0 and 1 results in the following equation:

$$risk(\Delta t | disease\ free\ at\ t) = r$$

This is an expression for the probability that within the interval Δt STR will be detected. The stricter (lower) r, the shorter Δt.

ESM Table 1: Details of costs

Cost item	Time (hour) (max - min)	Unit price (euro per hour)	Rate (euro) (max - min)	Reference
Screening	-	-	15.25 – 41.07	[2, 3]
Travel	0.17 – 1.50	9.46	1.61 – 14.19	[2]
Productivity loss	0.42 – 2.63	6.32	2.63 – 16.62	[4]

The unit price for travel cost was calculated based on a previous study [2].
The maximum screening cost was based on a tariff from the Organization for integrated medical diagnostics and advice for primary and secondary health care [3].
The minimum screening cost was based on a microcosting analysis (unpublished) with the following assumptions: €14.72 for primary and €23.51 for secondary care based on costs for 4 items, namely taking photo, assessment/processing/administration, overhead and medical depreciation. Based on expert opinion, 94% of patients were in primary care and 6% in secondary care [2].
The unit price for productivity loss was calculated based on the percentage of working people in a cohort of people with diabetes presented in a previous study, combined with information from the Dutch guideline on hourly wage rates [4].

ESM Table 2: Risk margins and corresponding screening intervals, number of delayed STR diagnoses and cost saving of personalized screening compared to annual screening

Risk margin (%)	Mean screening interval (month)	Delayed STR diagnoses, n, (% of all STR)	
		Slow STR progression assumption	Fast STR progression Assumption
0.0	6	0 (0.0)	0 (0.0)
0.5	8.1	2 (1.3)	3 (1.9)
0.9	10.7	7 (4.5)	8 (5.2)
1.0	11.3	9 (5.8)	9 (5.8)
1.5	14.7	12 (7.7)	13 (8.4)
**2.0**[1]	**18.4**	**15 (9.7)**	**18 (11.6)**
2.5	22.1	17 (11.0)	22 (14.1)
2.7	23.5	17 (11.0)	22 (14.1)
3.0	25.6	18 (12.3)	24 (15.5)
3.5	28.8	21 (13.6)	28 (18.1)
4.0	31.7	25 (16.1)	33 (21.3)

[1]Optimum risk margin for fast and slow STR progression assumption based on average cost saving per delayed STR diagnosis
STR: Sight-threatening retinopathy

ESM Table 3: Delay in diagnosing STR patients in the personalized model and the Dutch guideline

**Variable**	**Risk margin 2.0%**		**Dutch guideline**	
	Slow STR progression assumption	*Fast STR progression assumption*	*Slow STR progression assumption*	*Fast STR progression assumption*
Number of delayed STR diagnoses, (% out of 155)	15 (9.7)	18 (11.6)	28 (18.1)	32 (20.7)
Mean delay, (SD) in months	18.2 (14.5)	18.8 (15.5)	16.6 (5.9)	16.0 (5.7)
Median delay (IQR) in months	13.5 (6.1-26.2)	15.3 (6.1-28.4)	12.0 (12.0-24.0)	12.0 (12.0-24.0)

STR: Sight-threatening retinopathy; IQR: inter-quartile range; SD: standard deviation

ESM Table 4: The retinopathy grade at baseline for delayed STR diagnoses

Grade at baseline	Risk margin 2.0%		Dutch guideline	
	Slow STR progression assumption	*Fast STR progression assumption*	*Slow STR progression assumption*	*Fast STR progression assumption*
Grade 0	14	16	29	33
Grade 1	2	2	0	0
Grade 2	0	1	0	0

STR: Sight-threatening retinopathy

ESM Table 5: Total costs, saving, ICER and number of delayed STR diagnoses for different strategies with the slow progression assumption

Item	Healthcare perspective	Societal perspective
Total costs – annual screening (1000 €)	682 (433, 966)	1018 (721, 1336)
Total costs – Personalized screening (1000 €)	361 (228, 510)	539 (380, 717)
Total costs – Dutch guideline screening (1000 €)	310 (196, 437)	472 (325, 610)
Saving per patient per year - Personalized compared to annual (€)	11.4 (7.5, 16.5)	17.2 (12.4, 22.9)
Saving per patient per year – Dutch guideline compared to annual (€)	13.2 (8.8, 19.1)	19.9 (14.3, 26.7)
ICER saving per delayed STR diagnosis - Personalized compared to annual (1000 €)	24 (13, 40)	34 (19, 55)
ICER saving per delayed STR diagnosis – Dutch guideline compared to annual (1000 €)	14 (8, 22)	21 (13, 30)
Number of delayed STR diagnoses – Personalized screening	15.2 (9.5, 21.0)	15.2 (9.5, 21.0)
Number of delayed STR diagnoses – Dutch guideline screening	27.7 (20.0, 36.4)	27.7 (20.0, 36.4)

STR: sight-threatening retinopathy; 95% confidence interval in parentheses; ICER: incremental cost-effectiveness ratio

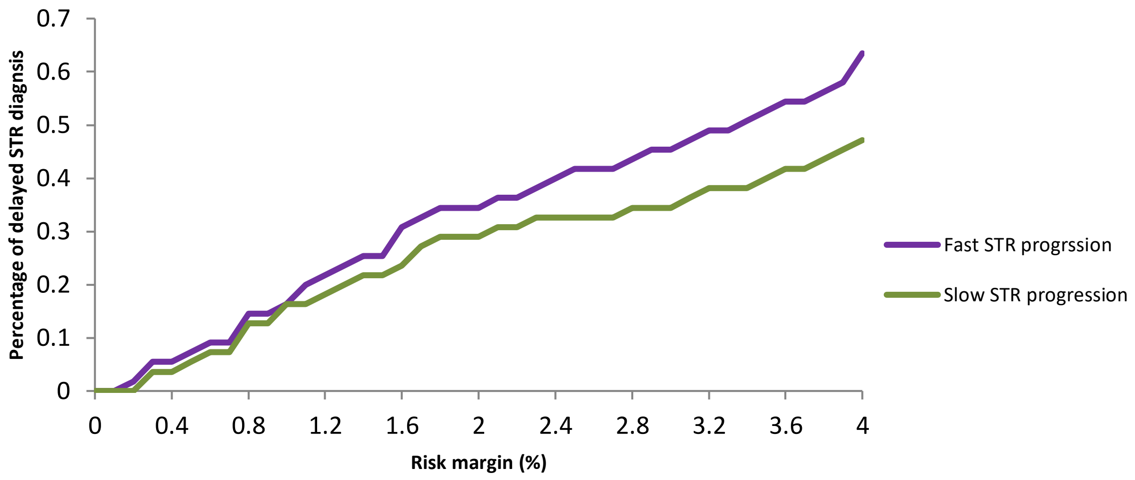

ESM Figure 1: The percentage of delayed STR diagnoses for different risk margins
STR: Sight-threatening retinopathy

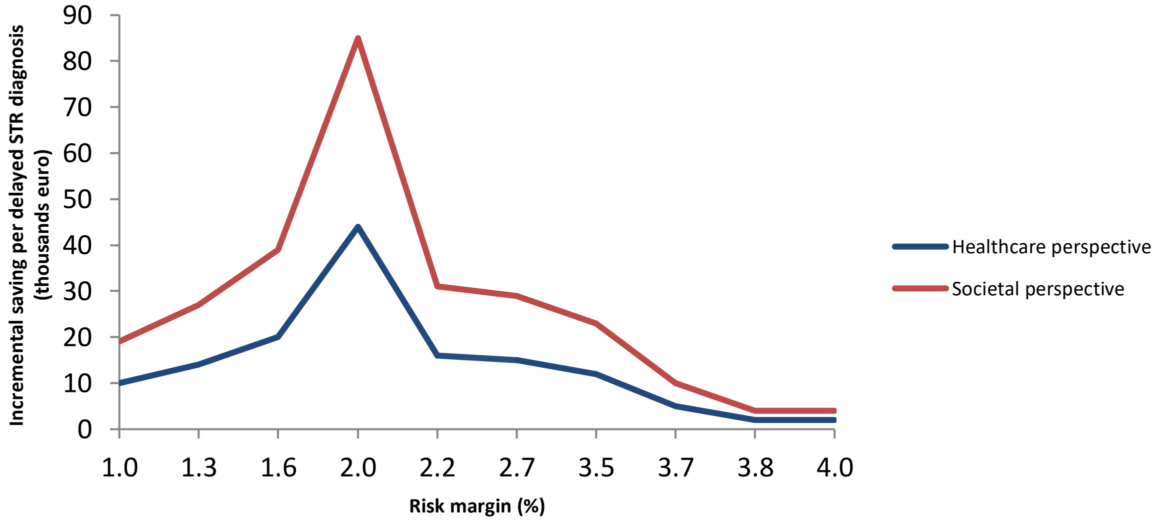

ESM Figure 2: Incremental saving per delayed STR diagnosis from a healthcare and a societal perspective for fast STR progression
STR: Sight-threatening retinopathy; RM: risk margin

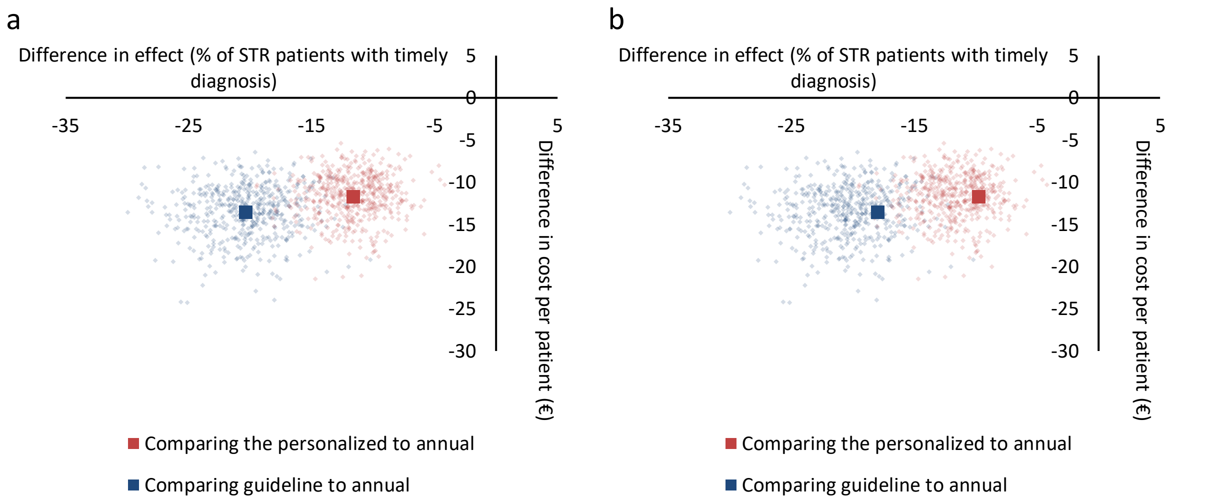

ESM Figure 3: Cost-effectiveness plane with 1000 bootstrapping simulations from the healthcare perspective; (a) fast STR progression and (b) slow STR progression
STR: Sight-threatening retinopathy

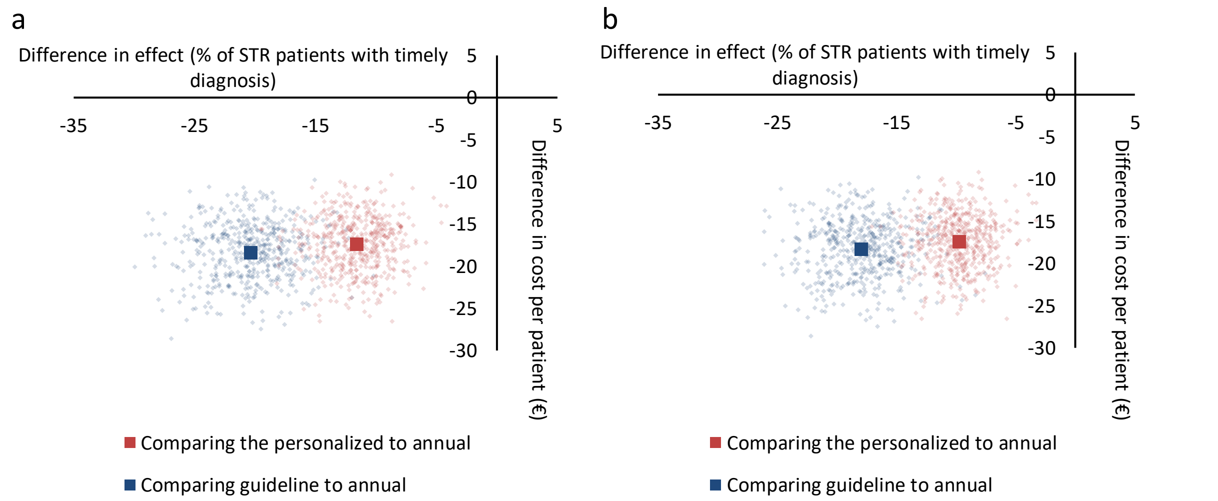

ESM Figure 4: Cost-effectiveness plane with 1000 bootstrapping simulations from the societal perspective; (a) fast STR progression and (b) slow STR progression
STR: Sight-threatening retinopathy

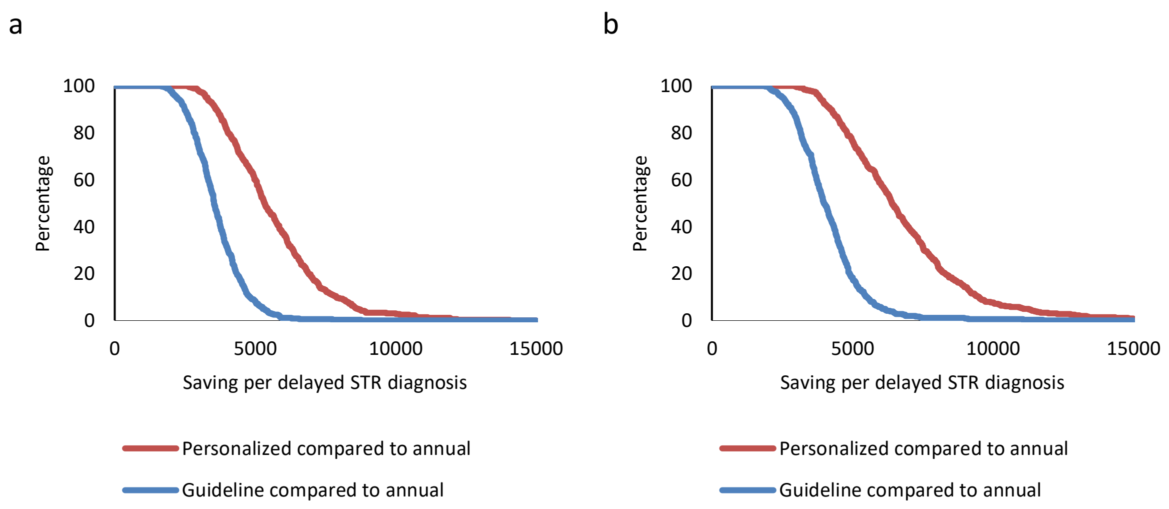

ESM Figure 5: Cost-effectiveness acceptability curve with 1000 bootstrapping simulations from healthcare perspective; (a) fast STR progression and (b) slow STR progression

STR: Sight-threatening retinopathy

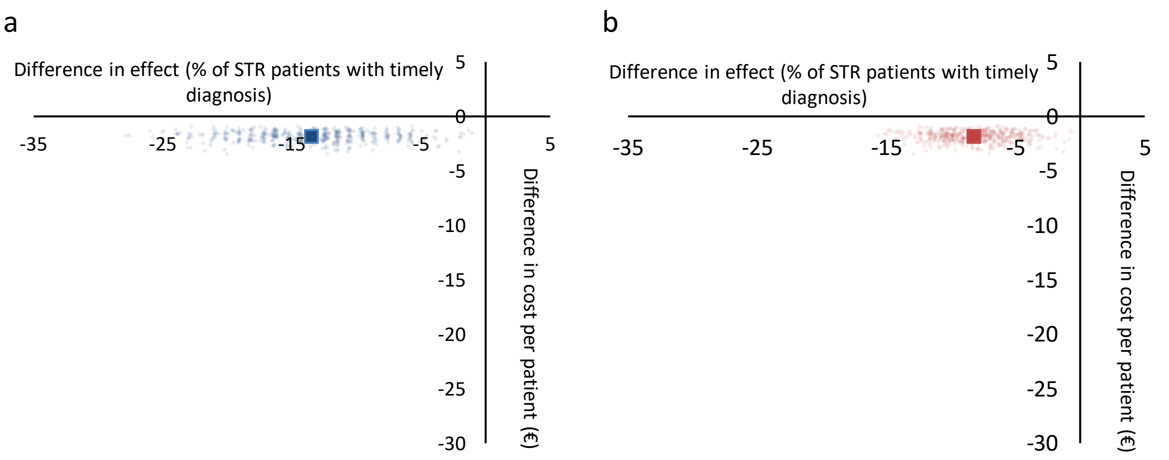

ESM Figure 6: Cost-effectiveness plane with 1000 bootstrapping simulations for comparing Dutch guideline with personalized screening from the healthcare perspective; (a) fast STR progression and (b) slow STR progression

STR: Sight-threatening retinopathy

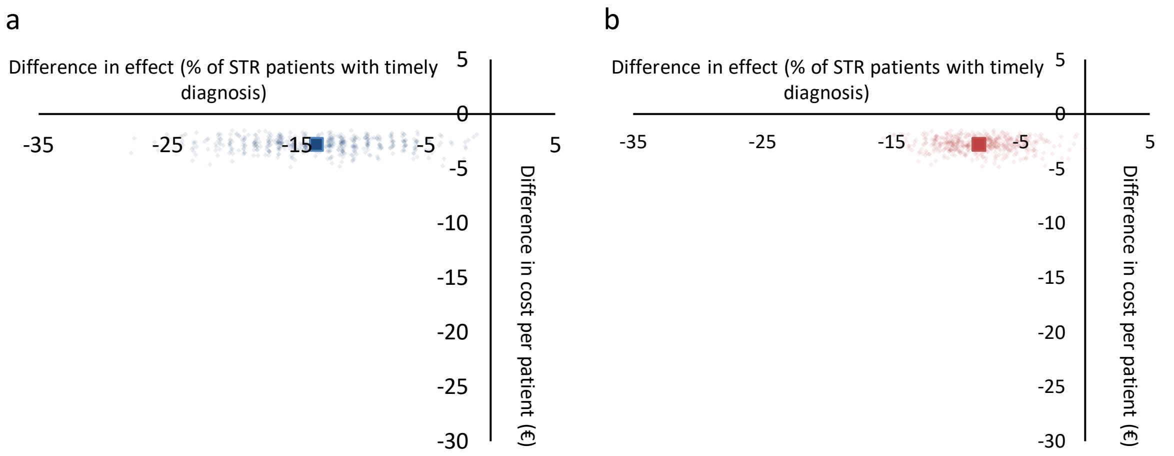

ESM Figure 7: Cost-effectiveness plane with 1000 bootstrapping simulations for comparing Dutch guideline with personalized screening from the societal perspective; (a) fast STR progression and (b) slow STR progression
STR: Sight-threatening retinopathy

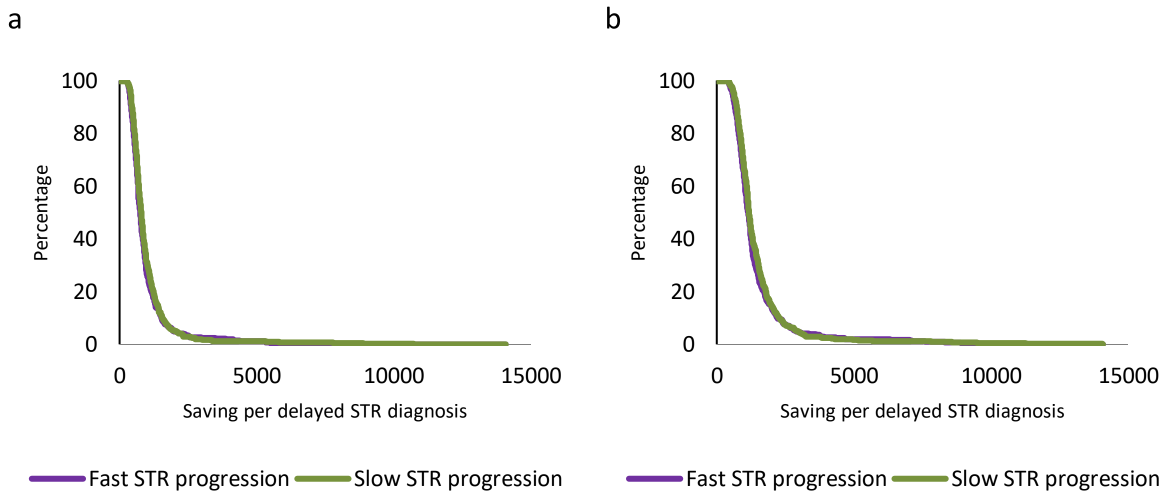

ESM Figure 8: Cost-effectiveness acceptability curve with 1000 bootstrapping simulations for comparing Dutch guideline with personalized screening; (a) from healthcare perspective and (b) societal perspective
STR: Sight-threatening retinopathy

References

[1] Aspelund T, Þórisdóttir Ó, Ólafsdottir E, et al. (2011) Individual risk assessment and information technology to optimise screening frequency for diabetic retinopathy. Diabetologia 54(10): 2525-2532. 10.1007/s00125-011-2257-7

[2] Heijden AAvd, Nijpels G, Baan CA, et al. (2016) An innovative strategy for diagnostic fundoscopy in diabetes patients reduces care use and costs compared to usual care. Available from https://www.zonmw.nl/nl/onderzoek-resultaten/doelmatigheidsonderzoek/programmas/project-detail/doelmatigheidsonderzoek/an-innovative-strategy-for-diagnostic-fundoscopy-in-diabetespatients-reduces-care-use-and-costs-com/. Accessed March 2020

[3] Organization for integrated medical diagnostics and advice for primary and secondary health care availabe from: https://www.certe.nl/. Accessed March 2020

[4] Janssen L, Hiligsmann M, Elissen A, et al. (2020) Burden of disease of type 2 diabetes mellitus: cost of illness and quality of life estimated using the Maastricht Study. Diabetic Medicine
